# Transcription factor expression as a predictor of colon cancer prognosis: a machine learning practice

**DOI:** 10.1186/s12920-020-00775-0

**Published:** 2020-09-21

**Authors:** Jiannan Liu, Chuanpeng Dong, Guanglong Jiang, Xiaoyu Lu, Yunlong Liu, Huanmei Wu

**Affiliations:** 1 Depart of BioHealth Informatics, School of Informatics and Computing, Indiana University-Purdue University Indianapolis, Indianapolis, IN USA; 2 Center for Computational Biology and Bioinformatics, Indiana University School of Medicine, Indianapolis, IN USA; 3 Department of Medical and Molecular Genetics, Indiana University School of Medicine, Indianapolis, IN USA; 4 Temple University College of Public Health, Philadelphia, PA USA

**Keywords:** Colon cancer, Transcription factor, Machine learning, Cancer prognosis

## Abstract

**Background:**

Colon cancer is one of the leading causes of cancer deaths in the USA and around the world. Molecular level characters, such as gene expression levels and mutations, may provide profound information for precision treatment apart from pathological indicators. Transcription factors function as critical regulators in all aspects of cell life, but transcription factors-based biomarkers for colon cancer prognosis were still rare and necessary.

**Methods:**

We implemented an innovative process to select the transcription factors variables and evaluate the prognostic prediction power by combining the Cox PH model with the random forest algorithm. We picked five top-ranked transcription factors and built a prediction model by using Cox PH regression. Using Kaplan-Meier analysis, we validated our predictive model on four independent publicly available datasets (GSE39582, GSE17536, GSE37892, and GSE17537) from the GEO database, consisting of 925 colon cancer patients.

**Results:**

A five-transcription-factors based predictive model for colon cancer prognosis has been developed by using TCGA colon cancer patient data. Five transcription factors identified for the predictive model is HOXC9, ZNF556, HEYL, HOXC4 and HOXC6. The prediction power of the model is validated with four GEO datasets consisting of 1584 patient samples. Kaplan-Meier curve and log-rank tests were conducted on both training and validation datasets, the difference of overall survival time between predicted low and high-risk groups can be clearly observed. Gene set enrichment analysis was performed to further investigate the difference between low and high-risk groups in the gene pathway level. The biological meaning was interpreted. Overall, our results prove our prediction model has a strong prediction power on colon cancer prognosis.

**Conclusions:**

Transcription factors can be used to construct colon cancer prognostic signatures with strong prediction power. The variable selection process used in this study has the potential to be implemented in the prognostic signature discovery of other cancer types. Our five TF-based predictive model would help with understanding the hidden relationship between colon cancer patient survival and transcription factor activities. It will also provide more insights into the precision treatment of colon cancer patients from a genomic information perspective.

## Background

Colon cancer is the sixth in men and the fifth in women the most common cause of cancer-related death globally [1]. In the United States, colon cancer is estimated to have 135,430 newly diagnosed cases and result in 50,260 deaths in 2017, accounting for 9% of cancer deaths [1]. Colon cancer is a complex disease with many risk factors, such as genetics, lifestyles, and dietary habits. Among them, inherited gene mutation, which can pass through family members, is one critical factor to increase one’s colon cancer risk. A common colon cancer feature is the intra-cancer heterogeneity, which makes patients distinctive from each other in clinical presentations and responses to treatment. Colon cancer treatments should be tailored based on the individual’s risk factors and genetic factors.

The inherited colon cancers can be broadly classified into two categories: familial adenomatous polyposis (FAP) and hereditary nonpolyposis colorectal cancer [2]. Molecular features in the genomics level play an essential role in treatment decision making and will continue providing more insights for pathological classification and tailored treatment for colon cancer. Proper colon cancer classification will significantly improve the survival rate, but hinders considerably by limited available prognosis assays.

Among the genetic factors, transcription factors (TFs) play a vital role in most important cellular processes, such as cell development, response to inner and outer environment change, cell cycle controls, and carcinogenesis. TFs are proteins that control the transcription of fragment DNA to messenger RNA by binding to specific DNA regions [3]. Their functions are to regulate, turn on and off genes to make sure that genes expressed in the right cell at the right time and in the right amount throughout the life of the cell and the organism [4]. For example, the NF-κB comprises a family of five TFs that form distinct protein complexes, which bind to consensus DNA sequences at promoter regions of responsive genes regulating cellular processes. NF-κB signaling and its mediated transcription play a critical role in inflammation and colorectal cancer development [5]. STAT3 is reported constitutively activated in colon-cancer-initiating cells and play a significant role in colon cancer progression [6]. FOXM1 was another TF that had been reported to be a key regulator of cell cycle progression, inflammation, tumor initiation and invasion [7].

In the past two decades, many researchers have implemented machine learning (ML) methods in the discovery and validation of cancer prognosis, especially after the population of High Throughput Technologies (HTTs) [8]. Recently, Long Nguyen Phuoc, et al. [9] developed a novel prognosis signature in colorectal cancer (CRC) by implementing several ML methods on public available CRC omics data. Their results demonstrated that the random forest method outperformed other ML methods they tried. Some researchers focused on microRNAs to find cancer prognosis signatures. Fatemeh Vafaee, et al. [10] proposed a prognostic signature of colorectal cancer comprising 11 circulating microRNAs. They also tested several different ML methods including RF and AdaBoost in their study. Their performance of the proposed prognostic signature was confirmed by an independent public dataset. Similarly, Jian Xu, et al. [11] developed a 4-microRNA expression signature for colon cancer patients by using the data from The Cancer Genome Atlas (TCGA). Their study showed that this 4-microRNA signature might play an important role in cancer cell growth after anti-cancer drug treatment. In 2016, Guangru Xu, et al. [12] discovered a 15-gene signature that could effectively predict the recurrence and prognosis of colon cancer using a Support Vector Machine (SVM) algorithm. Their study pointed out that some genes in this signature might be an indicator of new therapeutic targets. Although these previous studies implemented machine learning methods on the discovery of cancer prognosis signatures, the crucial role of TFs has not been sufficiently addressed in cancer prognosis signature development.

The goal of our study is to identify the fundamental transcript factors, which are associated with clinical outcomes of colon cancer patients, by implementing an innovative cancer prognosis signature discovery process that combines the random forest algorithm with classic Cox Proportional Hazard (Cox PH) method. Our study will emphasize on only using TFs expression data to conduct prognostic analysis and we will provide a new perspective on how we can better use gene expression profiles to conduct prognostic research. By using proposed workflow, a TFs based prediction model has been successfully developed for colon cancer prognosis. The prediction power of our model is validated on hundreds of colon cancer patient samples available in the GEO database [13]. Our TF-based colon cancer prognosis prediction model can be used for a better classification of colon cancer patients in survival. Successful findings of this study will shed lights on understanding the mechanisms of the underlying colon cancer development and metastasis.

## Methods

### Data sources

In this study, we are using the expression data of TFs from two public resources. One is TCGA colon cancer (COAD) dataset, which can be downloaded from UCSC Xena (http://xena.ucsc.edu) [14] for both the expression dataset and the clinical data of patients. There are 497 samples in the COAD dataset, including 456 primary cancer tissue samples and 41 adjacent normal tissue samples. The downloaded TCGA level 3 RNAseq data is in the log2(counts + offset) format. The TCGA COAD dataset is used as the training set in this study to build the predictive model for the colon cancer prognosis. Only patients carrying a primary tumor with the overall survival times and events were included in the training dataset. Then we further filtered the dataset by excluding patients who have missing information in cancer stage and other clinical information including sex and age. Finally, 435 patients with primary cancer tissue information were remaining in the training TCGA dataset,

The second public expression data resource is the microarray data from GEO database, which will be used to validate our prediction model. We chose four Affymetrix Human Genome U133 Plus 2.0 Array microarray study as validation datasets. The accession numbers, sequencing platform information, and sample sizes of each GEO dataset used in this study were listed in Table 1. The respective clinical data were retrieved from published literature. The GEO dataset also filtered similarly to the TCGA COAD dataset with the survival events and times. In the end, the total number of GEO samples we used for prediction model validation is 1584. Before performing further analysis, the Affymetrix microarray data were normalized using the Robust Multi-array Average (RMA).

**Table 1:** Summary of the general clinicopathologic characteristics of patients in both training and testing datasets

Characteristic	TCGA (*N* = 435)	GSE39582 (*N* = 563)	GSE17536 (*N* = 177)	GSE37892 (*N* = 130)	GSE17537 (*N* = 55)
	N (%)	N (%)	N (%)	N (%)	N (%)
Age (years)					
Median	66	68	66	68	62
Range	31–90	22–97	26–92	22–97	23–94
< 65	166 (38.2)	211 (37.5)	78 (44.1)	54 (41.5)	32 (58.2)
≥ 65	269 (51.8)	351 (62.3)	99 (55.9)	76 (58.5)	23 (41.8)
Sex					
Male	202 (46.4)	309 (54.9)	96 (54.2)	69 (53.1)	26 (47.3)
Female	233 (53.6)	253 (44.9)	81 (45.8)	61 (46.9)	29 (52.7)
T Status^a^					
T1–2	86 (19.8)	56 (9.9)	*NA*	*NA*	*NA*
T3–4	345 (79.3)	483 (85.8)	*NA*	*NA*	*NA*
N Status^a^					
N0	254 (58.4)	299 (53.1)	*NA*	*NA*	*NA*
N1	100 (23.0)	133 (23.6)	*NA*	*NA*	*NA*
N2	78 (17.9)	98 (17.4)	*NA*	*NA*	*NA*
M Status^a^					
M0	318 (73.1)	479 (85.1)	*NA*	*NA*	*NA*
M1	60 (13.8)	61 (10.8)	*NA*	*NA*	*NA*
MX	47 (10.8)	2 (0.4)	*NA*	*NA*	*NA*
Stage					
I	73 (16.8)	32 (5.7)	24 (13.6)		4 (7.3)
II	167 (38.4)	262 (46.5)	57 (32.2)	73 (56.2)	15 (27.3)
III	124 (28.5)	204 (36.2)	57 (32.2)	57 (43.8)	19 (34.5)
IV	60 (13.8)	60 (10.7)	39 (22)		17 (30.9)

^a^*T status* Describes the size of primary tissue and whether it has invaded nearby tissue, *N status* Describes nearby lymph nodes that are involved, *M status* Describes distant metastasis

As shown in Table 1 for the summary of the training and testing datasets, there are substantial similarities upon patient diagnosed age, gender and in the AJCC staging level. The consistency in the pathology levels renders convincing for further analysis without bias or overfitting.

### Workflow of the study

**Fig. 1:**
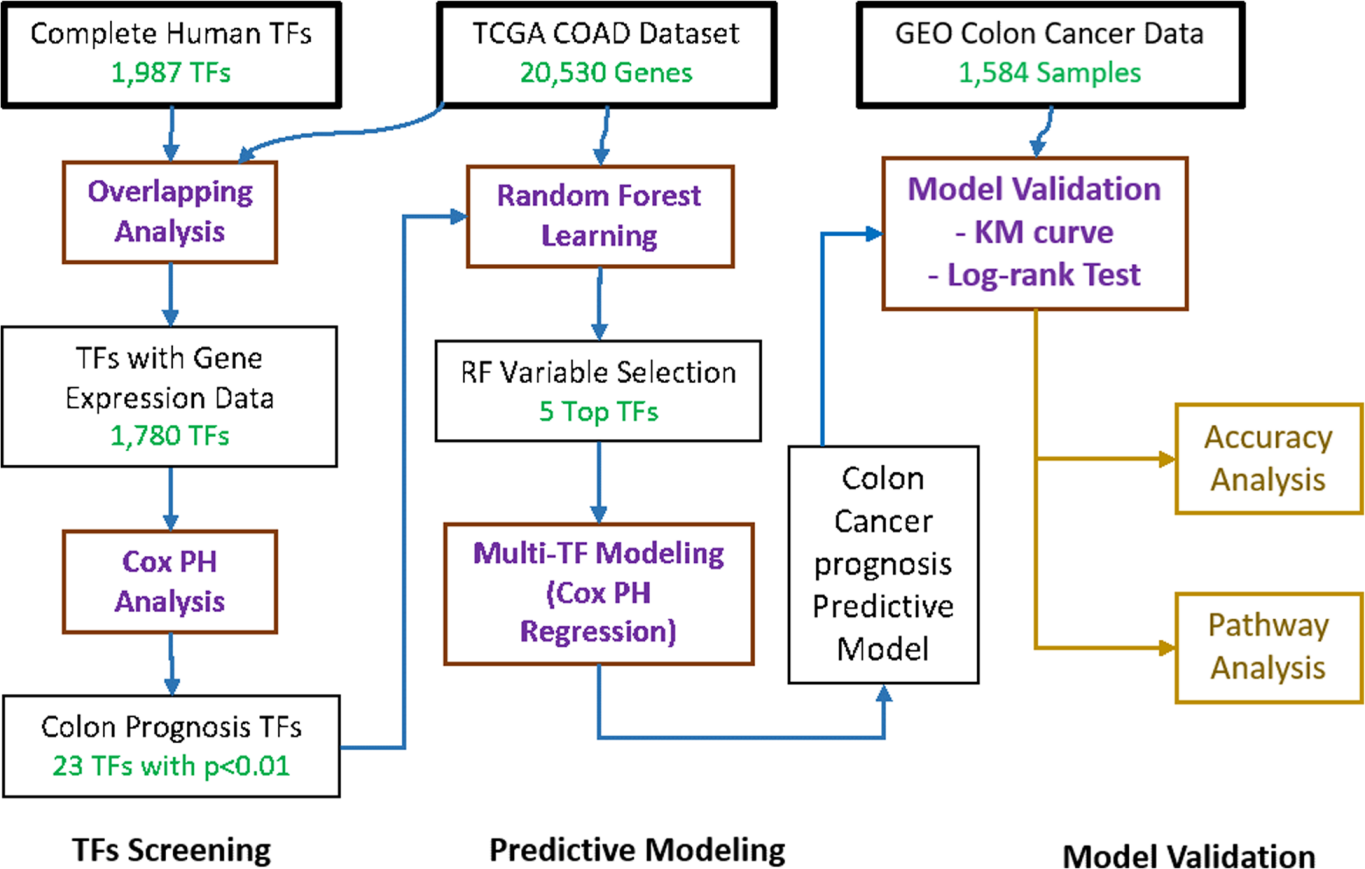
Workflow of this study. (TFs Screening, Predictive Modeling; Model Validation)

The overall workflow of our study is demonstrated in Fig. 1, which can be classified into three stages: TFs Screening, Predictive Modeling, and Model Validation. In Stage 1, we first identified a complete list of human TFs with official annotation from previous publications. Since not all the human TFs have the expression data in TCGA COAD dataset, the overlapped genes between TCGA COAD dataset and the complete list of TFs identified. Among the overlapping TFs, we further narrow down the numbers of TFs by the Cox PH Model analysis, which resulted in a limited set of TFs. Cox PH model is a widely used and performance proved statistical model in prognostic signature construction [8].

In Stage 2, since there are still too many colon prognosis TFs (more than 20 TFs), we need to decrease the final prognosis TFs to build a valid and good performance prognosis signature. The ensemble learning method, random forest method, is performed to refine further and reduce the TFs. Based on the RF training results, the most significant TFs are selected based on the top feature importance of RF. With the final TF list, we trained a predictive model for colon cancer prognosis using Cox PH regression.

Stage 3 is the validation of the predictive model. First, the prediction power is tested by accuracy analysis. Furthermore, the predictive model is validated on colon cancer datasets, collected from GEO database, including 925 samples from 4 studies. The Gene Set Enrichment Analysis (GSEA) [15] was also conducted to obtain further insights into our prediction model in the pathway level.

### Details on the variable selection and survival analysis methods

In Stage 1 of the variable selection, we used the univariate Cox PH model in the statistical environment R (v3.4), the association between expression profiles of TFs and the overall survival of patients was calculated to identify the prognostic ones. Any TF with a *p*-value less than 0.01 was considered statistically significant and used for further investigation.

In Stage 2 of refining variable selection, we performed RF methods for variable selection given that RF can be used for both classification problems and regression problems. RF [16] is an ensemble algorithm that use a bagging method to combine the multiple decision trees. It draws a set of samples from the whole dataset with replacement to feed the decision tree. After one decision tree has been trained, another sample set will be drawn from the whole dataset to train another decision tree. The process is repeated in the RF algorithm until the desired number of decision trees are trained. The final output of the prediction RF model can be the average of each decision tree’ output. In cancer prognosis signature discovery practice, RF is a performance proved method [9, 10, 17]. In our study, the *randomForestSRC* for survival package [18] was used to measure the importance of each variable’s contribution to the overall survival of colon cancer patients. This package uses minimal depth variable selection. The algorithm is the termed RSF-Variable Hunting [19]. It exploits maximal subtrees for effective variable selection in survival data scenarios. In our implementation, the parameters used in the feature selection RF model were ntree = 1000 and nstep = 5.

In Stage 3, for the validation of the predictive model, the Kaplan-Meier (KM) curve [20] was used to estimate the difference in the survival between high and low risk groups in validation datasets. The log-rank test [21] was conducted to test the significance of the difference between subgroups since the log-rank test is a very robust statistical method to test important differences between two groups and is widely used in clinical trial experiments.

## Results

### The results of identifying the potential prognostic transcription factors

The complete list of 1987 human TFs was downloaded based on the census of human TFs from the Nature Review Genetics paper by Vaquerizas, Juan M., et al. [22]. Among the listed human TFs, 1834 of them have gene symbols annotations. After mapping to TCGA COAD dataset, only 1780 TFs have gene expression data in TCGA COAD dataset, which were included in this study.

The univariate Cox PH regression was applied to the gene expression profiles for the overlapping 1780 TFs and the patient clinical data in TCGA colon cohort, to identify the TFs, which are associated with the survival of the patients and have the potential using as prognostic markers. Those TFs with *p* ≤ 0.01 were kept for further analysis (The selected 23 TFs are listed in Supplementary Table S1).

### Results on building the multi-TF predictive model

**Fig. 2:**
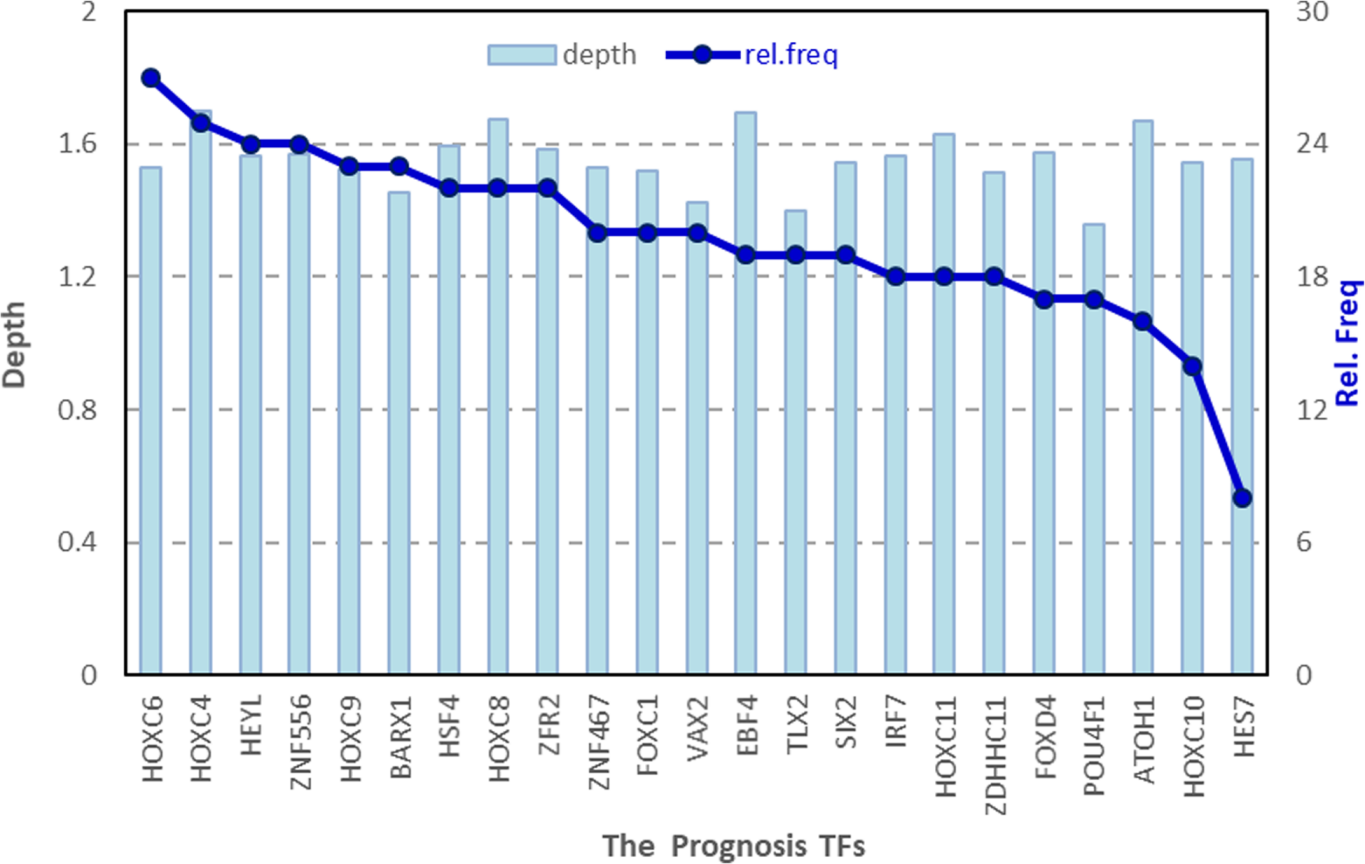
The RF results of the prognosis TFs for the Depth and relative frequency

To identify the minimum subset of TFs that can still achieve a good prediction of colon cancer survival, the 23 TFs from the Cox PH regression model were further evaluated with a random forest algorithm, *randomForestSRC*. In the *randomForestSRC* variable hunting mode, top P ranked variables will be selected, P is the average model size and variables are ranked by frequency of occurrence. In our study, five TFs (i.e.*,* HOXC9, ZNF556, HEYL, HOXC4, and HOXC6) were chosen for the final predictive model construction. The results of the algorithm is shown in Fig. 2. The parameters for random forest are ntree = 1000 and nstep =5.

To establish a multiple molecular based regression model, the multivariate Cox PH regression was trained with gene expression data using the five TFs and clinical variables from TCGA COAD dataset. The coefficients from the Cox model were then applied to a multivariate linear regression model. The risk score was calculated with the following formula:

$$ \mathrm{Risk}\ \mathrm{score}=0.139\ast \mathrm{HOXC}6-0.046\ast \mathrm{HOXC}4+0.165\ast \mathrm{HEYL}+0.106\ast \mathrm{ZNF}556-0.032\ast \mathrm{HOXC}9 $$

**Fig. 3:**
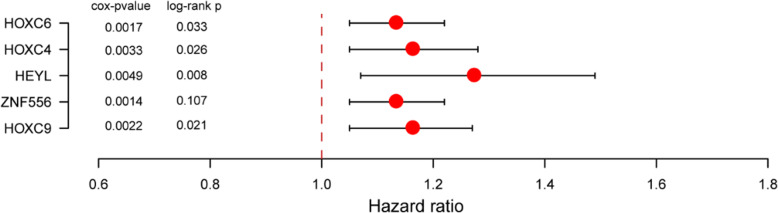
Information on five prognostic TFs finally selected for building the prediction model

The final coefficients of the model have been modified automatically to achieve better performance and to increase accuracy overall. Thus, the coefficients of HOXC9 and HOXC3 are adjusted to slightly below zero, which are much smaller than those positive coefficients. Then we performed the KM analysis and the log-rank test result over these five selected TFs. The results and the *p*-value from previous Cox PH analysis, along with the hazard ratio for each of these genes are summarized in Fig. 3. It can be seen that all selected 5 TFs has Cox *p*-value < 0.01, which indicates all these TFs are highly related to the overall survival of patients according to Cox PH analysis. For the log-rank p, only the ZNF556 has a *p*-value of 0.107, while all the other four have *p*-value < 0.05. According to the RF results, the importance of ZNF556 is ranked fourth in all 23 TFs with no significant difference with other TFs in maximum depth (Fig. 2), this qualifies the ZNF556 as one of the most important prognostic TFs. The Hazard ratios of all these five TFs are more than 1.0, indicating higher risks of colon cancer prognosis.

### Results on validation of the five-TF based prediction model

**Fig. 4:**
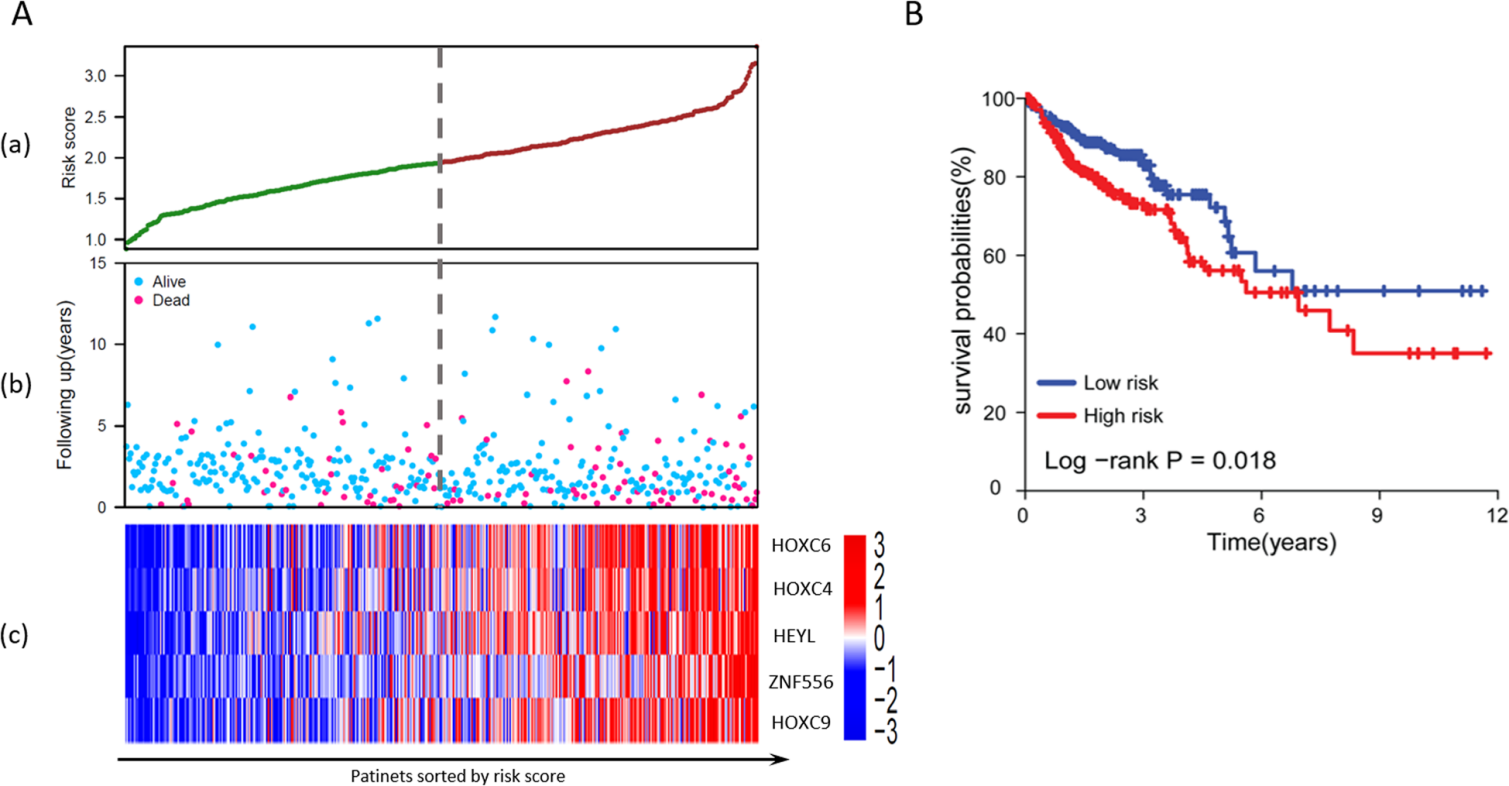
A multivariate linear regression model based on expression of five TFs. A. The patient survival (follow-up distribution) and selected genes expression profile, among with the calculated risk scores; B. The KM curve for predicted high-risk subgroup and low-risk subgroup using TCGA COAD dataset

Based on the median value of the predicted risks scores of all the patients in both the training and validation set, patients are classified into high-risk and low-risk subgroups. KM curve analysis and log-rank test were conducted to evaluate the performance of predicting power in colon cancer prognosis on TCGA COAD dataset. The results are shown in Fig. 4. The scatter plot (Fig. 4A(b)) shows the distribution of patients’ overall survival status. The red point indicates the patient belonging to a high-risk group while a blue point indicates the patient belonging to a low-risk group. From the scatter plot, we can observe that the red points are more concentrated in the lower part of the figure. This is an indication that high-risk patients have a shorter survival time comparing to low-risk patients. The heatmap (Fig. 4A(c)) shows that the five selected TFs in our predictive model were highly expressed in TCGA COAD dataset. Moreover, the KM curve (Fig. 4B) shows a distinctive survival difference between the high-risk and low-risk groups in a time span of more than 10 years. All these results prove the prediction power of our predictive model on TCGA COAD dataset.

**Fig. 5:**
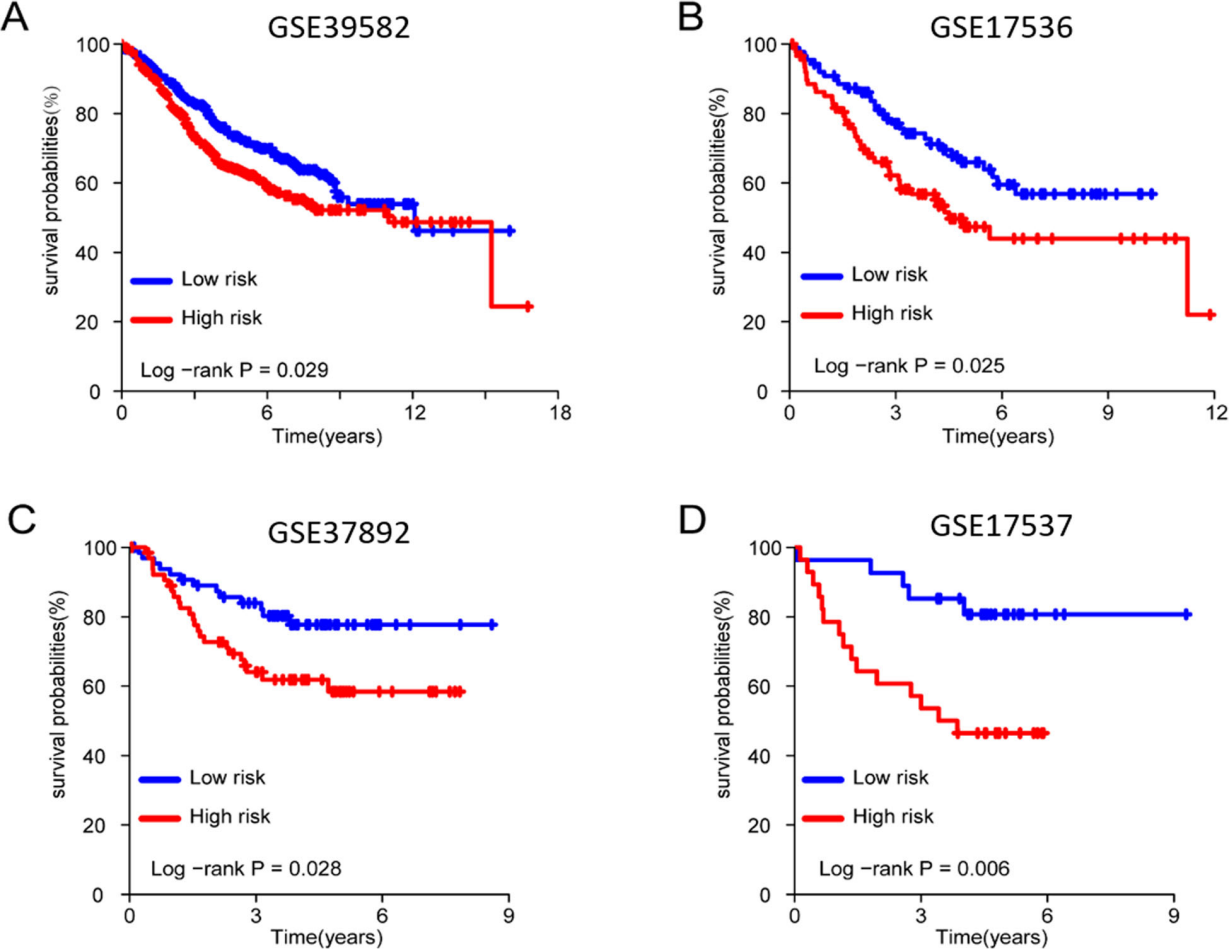
The KM curves of the overall survival probabilities for four independent validation datasets for predicted high-risk subgroups and low-risk subgroups

To test the five-TF based signature as colon cancer survival predictor, we further validated the predictive model on another four independent microarray datasets with a total of 1584 samples for GEO with GSE39582 (*n* = 563), GSE17536 (*n* = 177), GSE37892 (*n* = 130) and GSE17537 (*n* = 55). The risk score of each patient in validation dataset was calculated by using the same formula established with TCGA training dataset. The same coefficients were utilized to assign weight to each of the selected TF. By using the same median cutoff strategy to divide patients to the high-risk and low-risk groups, the KM curve analysis shows the consistent patterns with the TCGA COAD dataset. Patients in the high-risk group have a significantly shorter survival time than patients in the low-risk group (Fig. 5a–d), which suggests the clinical robustness among multiple centers. Therefore, our five-TF based signature is proved to be a robust predictor for colon cancer survival.

### Results on pathway analysis

**Fig. 6:**
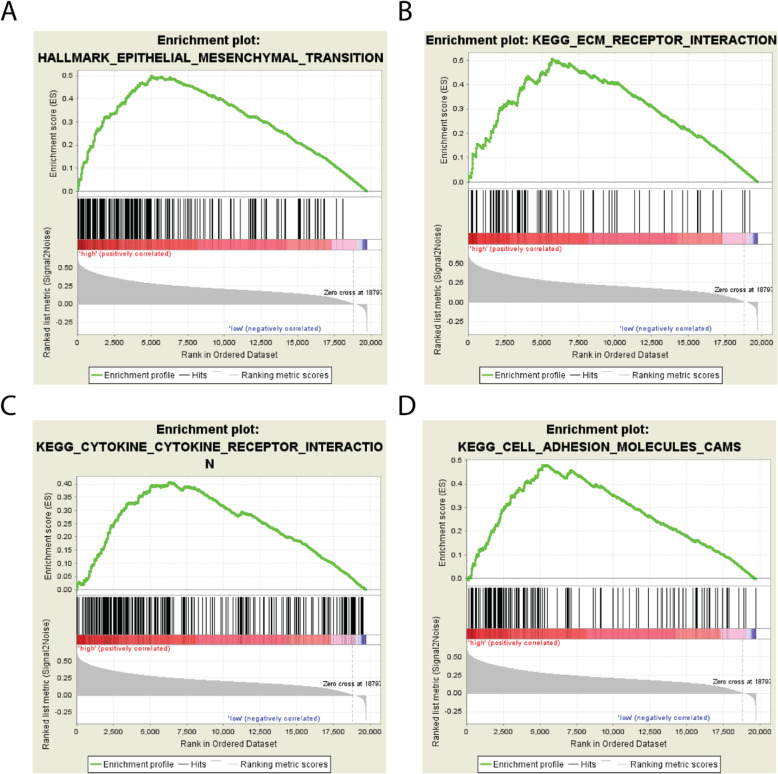
Enrichment plots for the top four enriched gene pathways according to the GSEA results. GSEA is performed on TCGA COAD dataset

The Gene Set Enrichment Analysis (GSEA) [15] was conducted to investigate the biological function of this five-TF based signature, including its molecular function and gene-gene network. GSEA is performed on the TCGA COAD dataset with predicted high-risk subgroup versus low-risk subgroup. In conducting the GSEA study, the reference gene pathway database is the Kyoto Encyclopedia of Genes and Genomes (KEGG) pathway database [23]. The GSEA number of permutations is set to be 1000, and the phenotype labels are determined according to whether a patient is in the high-risk subgroup or the low-risk subgroup. As illustrated in Fig. 6, the GSEA results showed that several cancer-related pathways were alternated in patients with high-risk scores, such as the pathways for the Epithelial-mesenchymal transition, the ECM receptor interaction, the cytokine-cytokine receptor interaction, and the cell adhesion molecules (Fig. 5a-d). Taken together these findings, it’s indicated that the five TFs in our model may highly associate with tissue morphogenesis, intercellular regulations and cell adhesion. By affecting these cell processes, these TFs may promote the tissue malignant then result in a poor overall survival rate of colon cancer patients.

## Discussions

We implemented an innovative machine learning approach for signature variables, which combines the Cox PH method with the random forest algorithm. Our signature selection process can find the minimum subset of TFs to build the prognosis prediction model with satisfying performance. A five-TF predictive model was developed by training the classifiers on TCGA COAD dataset. The trained multivariable linear predictive model was validated with multiple datasets from the GEO database.

Three out of the five selected genes, i.e.*,* HOXC4, HOXC6, and HOXC9, belong to the *homeobox* family of genes. The homeobox genes are highly conserved TF family and play an essential role in morphogenesis in all multicellular organisms. Dysregulation of HOX gene expression implicated as a factor in malignancies, and up-regulation has been observed in malignant prostate cell lines and lymph node metastases [24]. HOXC6 was also reported to be overexpressed in colorectal cancer tissue, and highly correlated with poor survival outcome and acts as a significant prognostic risk factor [25].

For the other two genes selected in our predictive model, HEYL belongs to the hairy and enhancer of split-related (HESR) family of basic helix-loop-helix (bHLH)-type transcription factor. A recent study shows that HEYL may be a tumor suppressor of liver carcinogenesis through upregulation of P53 gene expression and activation of P53-mediated apoptosis [26]. ZNF556 belongs to zinc finger protein (ZNF) family. Despite the large size of ZNF gene family, the number of disease-linked genes in this family is very small [27]. To the best of our knowledge, the research on ZNF556 related to cancer is very limited. Therefore, our study provided new insight on potential relationships between overexpression of ZNF556 and the development of colon cancer.

Our study also showed that by using TFs to build a predictive signature for colon cancer prognosis is practical. The prediction power of the model is promising. Intuitively, the TFs have the overall control on the gene expressions in cells so that a TF-based predictive model should be able to indicate the different gene expression levels in some cancer types with high accuracy.

Our innovative signature discovery process can potentially be extended on other cancer types such as breast cancer or lung cancer. It will be interesting to carry out studies on whether these five TFs used by our model have tissue specific expression patterns in colon cancer. Moreover, by conducting downstream analysis such as gene regulation network analysis, we can probably identify genes that are regulated by our five TFs, these downstream genes can be potentially added to the prediction model to add more robustness to our model. Another future study is to examine the performance of combining traditional statistical methods, such as Cox PH, with other machine learning methods, such as the artificial neural network (ANN) [28], to select potential prognostic TFs or other signatures for different types of cancer.

## Conclusion

We have successfully identified a five-TF signature and built a predictive model for colon cancer prognosis signature with the selected five TFs by using a machine learning approach. Our five-TFs based linear model was validated on hundreds of publicly available patient data from the GEO database. The results showed that our model has a good predicting power in predicting colon cancer overall survival. Our predictive model and its biological functions would provide more insights in the precision treatment of colon cancer, which leads to further investigation on these five TF genes and their roles during the development of colon cancer at the molecular level.

## Supplementary information

**Additional file 1: Table S1.** The 23 transcript factors identified as prognostic features.

## Data Availability

TCGA COAD dataset can be downloaded from http://xena.ucsc.edu, GEO datasets used in this study can be downloaded from GEO database by using access numbers: GSE39582, GSE17536, GSE37892, GSE17537.

## Abbreviations

TF: Transcription factor
ML: Machine learning
RF: Random forest
TCGA: The cancer genome atlas
GEO: Gene expression omnibus
KEGG: Kyoto Encyclopedia of Genes and Genomes
Cox PH: Cox proportional hazard
KM: Kaplan-Meier
GSEA: Gene set enrichment analysis
