# Supplementary material for: Transcription factor expression as a predictor of colon cancer prognosis: a machine learning practice
Source: BMC Med Genomics. 2020 Sep 21;13(Suppl 9):135. doi: 10.1186/s12920-020-00775-0 (PMC7504662; doi:10.1186/s12920-020-00775-0)
Supplement: Supplementary file 1 — Additional file 1: Table S1. The 23 transcript factors identified as prognostic features. [file 12920_2020_775_MOESM1_ESM.docx]

**Table S1**. The 23 transcript factors identified as prognostic features

|  | Cox PH | | | Log-rank test | RandomForest | |
| --- | --- | --- | --- | --- | --- | --- |
| Gene Name | Hazard ratio | 95% CI | Cox PH Pval | Log-rank P-val | depth | rel.freq |
| ZFR2 | 1.3 | (1.12 - 1.51） | 0.0006 | 0.0565 | 1.585 | 22 |
| BARX1 | 1.17 | (1.07 - 1.29） | 0.0006 | 0.0222 | 1.452 | 23 |
| VAX2 | 1.25 | (1.09 - 1.43） | 0.0011 | 0.0209 | 1.426 | 20 |
| HOXC8 | 1.16 | (1.06 - 1.28） | 0.0013 | 0.0018 | 1.673 | 22 |
| ZNF556 | 1.13 | (1.05 - 1.22） | 0.0014 | 0.1065 | 1.569 | 24 |
| ZDHHC11 | 1.18 | (1.06 - 1.30） | 0.0017 | 0.0232 | 1.516 | 18 |
| HOXC6 | 1.13 | (1.05 - 1.22） | 0.0017 | 0.0325 | 1.527 | 27 |
| ATOH1 | 0.9 | (0.84 - 0.96） | 0.0021 | 0.0006 | 1.670 | 16 |
| HOXC9 | 1.16 | (1.05 - 1.27） | 0.0022 | 0.0207 | 1.526 | 23 |
| HSF4 | 1.26 | (1.09 - 1.47） | 0.0024 | 0.0256 | 1.596 | 22 |
| SIX2 | 1.13 | (1.04 - 1.22） | 0.0024 | 0.0192 | 1.544 | 19 |
| HOXC11 | 1.1 | (1.04 - 1.18） | 0.0025 | 0.0213 | 1.630 | 18 |
| TLX2 | 1.22 | (1.07 - 1.39） | 0.0029 | 0.0253 | 1.400 | 19 |
| HOXC4 | 1.16 | (1.05 - 1.28） | 0.0033 | 0.0256 | 1.700 | 25 |
| EBF4 | 1.27 | (1.08 - 1.49） | 0.004 | 0.0709 | 1.695 | 19 |
| HES7 | 1.18 | (1.05 - 1.33） | 0.0045 | 0.1886 | 1.556 | 8 |
| HEYL | 1.27 | (1.07 - 1.49） | 0.0049 | 0.008 | 1.564 | 24 |
| FOXC1 | 1.19 | (1.06 - 1.35） | 0.0049 | 0.0085 | 1.518 | 20 |
| HOXC10 | 1.12 | (1.03 - 1.22） | 0.0071 | 0.3153 | 1.542 | 14 |
| POU4F1 | 1.14 | (1.04 - 1.26） | 0.0078 | 0.0657 | 1.357 | 17 |
| IRF7 | 1.31 | (1.07 - 1.61） | 0.008 | 0.0343 | 1.562 | 18 |
| FOXD4 | 1.19 | (1.04 - 1.35） | 0.009 | 0.0276 | 1.574 | 17 |
| ZNF467 | 1.27 | (1.06 - 1.53） | 0.0091 | 0.2035 | 1.572 | 20 |
